# Tetanus and Diphtheria Toxoid-Containing Vaccine in Multiple Sclerosis Patients: A Real-World Prospective, Open-Label, Multi-Centre Study

**DOI:** 10.3390/vaccines13050451

**Published:** 2025-04-24

**Authors:** Alexander Winkelmann, Emil C. Reisinger, Katharina Boden, Christoph Metze, Uwe K. Zettl, Micha Loebermann

**Affiliations:** 1 Department of Neurology, Gehlsheimer Str. 20, D-18147 Rostock, Germany; uwe.zettl@med.uni-rostock.de; 2 Department of Tropical Medicine and Infectious Diseases, Schillingallee 36, D-18057 Rostock, Germany; emil.reisinger@uni-rostock.de (E.C.R.); micha.loebermann@med.uni-rostock.de (M.L.); 3 SYNLAB MVZ Thüringen, Ernst-Ruska-Ring 15–17, D-07745 Jena, Germany; katharina.boden@synlab.com; 4 Section of Neuroimmunology, Gehlsheimer Str. 20, D-18147 Rostock, Germany

**Keywords:** multiple sclerosis, tetanus vaccination, diphtheria vaccination, disease-modifying-drugs, immunogenicity, safety

## Abstract

**Objective**: To assess changes in disease activity in Multiple Sclerosis (MS) patients on various disease-modifying-drugs, as well as immunogenicity, safety and clinical tolerability following combined tetanus- and diphtheria-vaccination. **Methods**: We conducted a prospective, multicentre, non-randomised real-world observational study at specialised outpatient MS care centres in Germany. We enrolled multiple sclerosis patients receiving a combined tetanus- and diphtheria-vaccination who had a stable MS-treatment regimen for at least six months and had an indication for this vaccination. Serum samples were obtained before and four weeks after vaccination for specific antibody response. Antibody concentrations against vaccine antigens were measured in duplicate via ELISA. Subjects were followed for one year after immunisation. MS disease activity (EDSS and relapse rates) was evaluated at follow-up visits. Local and systemic adverse events were registered four weeks after vaccination. **Results**: In total, 72 MS patients received tetanus and diphtheria vaccination. The annualised relapse rates in the year after vaccination were comparable to the year before vaccination (0.39 vs. 0.37). During the study period, the EDSS score did not change significantly. The score was 2.0 and 2.2 in the two years prior to vaccination and 2.5 in the year following vaccination. No subjects experienced severe adverse events. However, 14 (19.4%) had local adverse events, and 10 (13.9%) had systemic reactions. Following vaccination, all subjects had protective antibody titres against tetanus- and diphtheria-toxoid. Geometric mean antibody titres of tetanus toxoid antibodies increased from 0.64 IU/mL to 2.23 IU/mL (*p* < 0.0001) and of diphtheria toxoid antibodies from 0.1 IU/mL to 0.45 IU/mL (*p* < 0.0001). **Conclusions**: Tetanus- and diphtheria vaccination proved to be safe and effective in MS patients in a real-world situation.

## 1. Introduction

Multiple Sclerosis (MS) is a chronic immune-mediated condition that primarily affects young adults, with both neuroinflammatory and neurodegenerative components. [1,2].

In the context of vaccination, there is uncertainty from both people with multiple sclerosis (PwMS) [3,4] and their treating physicians [5] about the potential risks of influencing the immune system through vaccination, particularly regarding the dysregulation of the immune system in the context of MS [5,6,7,8]. Furthermore, there is concern about the usefulness and effectiveness of vaccinations in PwMS undergoing disease-modifying therapies (DMT), particularly whether clinically relevant vaccination success can be achieved [9,10,11]. On the other hand, only a small proportion of PwMS in the RWS meet the inclusion and exclusion criteria required for disease-modifying-drug (DMD) approval studies [12]. Findings from randomised controlled trials (RCTs) on vaccination success in PwMS with a specific DMD [13,14,15,16] must be confirmed in RWS, as patient cohorts often differ in factors such as MS severity, age, disease duration, previous immunological therapies, comorbidities, and potential polypharmacy [10].

In recent years, the long-term prognosis of PwMS has improved significantly, which is mainly due to the increasing availability of highly effective immunotherapies. The mechanisms of action of DMD are specifically based on influencing immune cell interaction, changes in lymphocyte traffic, depletion of lymphocytes and/or lymphocyte replication [9]. However, these mechanisms of action also represent an increased risk of infectious diseases on the one hand and the risk of potential impairment of the patient’s vaccination success on the other hand.

Another aspect is that the impact of causes and consequences of infections on the long-term outcome in PwMS are often underestimated in terms of everyday relevance [17]. For example, infectious diseases pose a major risk of disease deterioration, which is associated with a significant number of hospital admissions, morbidity and mortality [18,19,20]. In PwMS, vaccination provides additional benefits by preventing bacterial or viral infections that may trigger MS relapses [21,22,23]. Several studies show that infections are among the three major causes of death in MS patients [24,25]. In this context, clinical studies on vaccination against infectious diseases in RWS contribute significantly to the knowledge of optimal management of PwMS, both with and without DMDs [26,27,28]. Vaccination is an effective strategy to prevent these infection-associated complications in MS.

Except for live vaccines, PwMS and their physicians are encouraged to follow national and international vaccination guidelines, as there is no evidence that vaccines worsen existing MS or trigger its onset [9,29,30,31]. Furthermore, the use of non-live combination vaccines, such as those for tetanus and diphtheria, may be associated with a decrease in relapse rates [32] and has not been linked to an increased risk of developing MS [33]. A recent study did not show a negative impact on MS disease activity following a live yellow-fever vaccine [34].

In recent international guidelines, the importance of vaccination in MS patients as a mainstay of patient care is highlighted [10,35,36,37,38,39]. In the clinical routine, patients and their treating physicians display hesitancy to vaccinations due to fear of disease exacerbation despite these recommendations [6,7], emphasising the need for further real-world evidence [12]. Tetanus-diphtheria (Td) vaccines are included in national vaccine recommendations, with recommended schedules varying across Europe. Booster doses are typically advised at 10-year intervals for adults [40]. Tetanus infections are rare in regions with high immunisation rates, but may occur in individuals with lower antibody titres [41,42].

However, both MS itself and immunomodulatory or immunosuppressive DMDs used to treat MS can impair the immune response to vaccines [6,9].

In this study, we aim to evaluate the safety, tolerability and immunogenicity of vaccination against tetanus and diphtheria in MS patients receiving various disease-modifying-drugs in a real-world setting with long-term follow-up. Moreover, results can contribute to addressing the question of vaccine hesitancy in both patients and physicians.

## 2. Materials and Methods

### 2.1. Subjects and Study Procedures

We conducted a prospective, multicentre, non-randomised observational study at specialised outpatient MS care centres in Germany. The study included patients with MS, aged 18 years and above, who were on unchanged disease-modifying treatment (DMT) for at least six months and had an indication for Td-vaccination. Criteria for exclusion were current MS relapse or other disease activity during the previous six months or general contraindications against these vaccinations.

All patients who chose to receive vaccination on a routine basis according to local vaccination recommendations [43] were offered to participate in this study. Baseline characteristics were collected along with details of MS disease (date of diagnosis, clinical course, current score in the Expanded Disability Status Scale (EDSS) and 3, 6, 12 and 24 months before vaccination, relapse rate in the last and second to last year before vaccination, and currently and previously prescribed DMT). The subjects received a single intramuscular dose of a licensed combined Td vaccination (either alone or in combination with pertussis or poliomyelitis) in the deltoid muscle of the non-dominant arm in an open-label manner. The study protocol did not influence the choice of the brand of the vaccine used.

Serum samples were obtained before and 4 weeks after vaccination for specific antibody response. Serology was performed in duplicate from samples stored at −80 °C using commercial enzyme-linked immune assays to detect IgG-antibodies directed against tetanus toxin (SERION ELISAclassic, Tetanus IgG, Institut Virion/Serion GmbH, Würzburg, Germany) and diphtheria toxin (SERION ELISAclassic; Diphtheria IgG; Institut Virion/Serion GmbH, Würzburg, Germany) according to the manufacturer’s recommendations. The Virion/Serion ELISA was performed using the automatic immunoassay analyser BEPIII (Siemens, Berlin, Germany), and extinction was read at 405 nm. According to WHO recommendations [42], subjects with tetanus antibody titres were rated as follows: <0.01 IU/mL unprotected, 0.01–0.1 IU/mL with insufficient protection, 0.11–1.0 IU/mL sufficient protection, >1.0 IU/mL long-lasting protection. Antibody titres directed against diphtheria were rated as follows: <0.01 IU/mL no protection, 0.01–<0.1 IU/mL minimal protection, 0.1–<1.0 IU/mL protection, ≥1.0 IU/mL long-term protection.

Subjects were followed for one year after immunisation, with follow-up visits after 1, 3, 6 and 12 months. After four weeks, reported local and systemic adverse events following vaccination (AEFI) were registered. At follow-up visits, changes in medical condition, medication or MS disease activity (EDSS and relapse rates) were evaluated.

### 2.2. Ethical Approval

The study was approved by the ethics committees at the participating study (Ethical committee Rostock HV 2010-0002). Prior to participation, all participants provided written informed consent. The study was conducted according to current principles of Good Clinical Practice. The trial was registered at ClinicalTrials.gov (NCT02275741).

### 2.3. Statistical Analysis

The safety and immunogenicity were analysed in the intention-to-treat population; all patients who entered the study and received a vaccination were evaluated.

All statistical analyses were performed using GraphPad Prism 9; version 9.5.1, GraphPad Software, Boston, MA, USA. Where appropriate, the Wilcoxon-rank test was used at a 95% confidence interval (CI) (this was based on a type 1 error probability (a) of 5%) to compare pre- and post-vaccination antibody titres. All reported *p*-values are two-sided; values of 0.05 or less were considered to indicate statistical significance.

## 3. Results

A total of 72 subjects, aged between 20 and 62 years, received vaccination and were included in this study. The baseline characteristics of the subjects are shown in Table 1. The majority of included subjects were female (75%). Most of the patients were affected by RR-MS (94%) and had a mean duration of disease of 7.7 ± 5.5 years. The mean EDSS score at vaccination was 2.2 ± 1.6. Two subjects with RR-MS received no DMT. Four patients with SP-MS were treated with intrathecal triamcinolone, cyclic glucocorticosteroid pulses, beta-interferon 1b, or glatiramer acetate. Overall, 58 patients received platform therapies (interferon beta or glatiramer acetate) and 9 had highly active DMT (natalizumab or fingolimod).

All subjects received a combined Td vaccination; among them, 37 received a combined vaccine with poliomyelitis and 35 in combination with pertussis.

**Figure 1:**
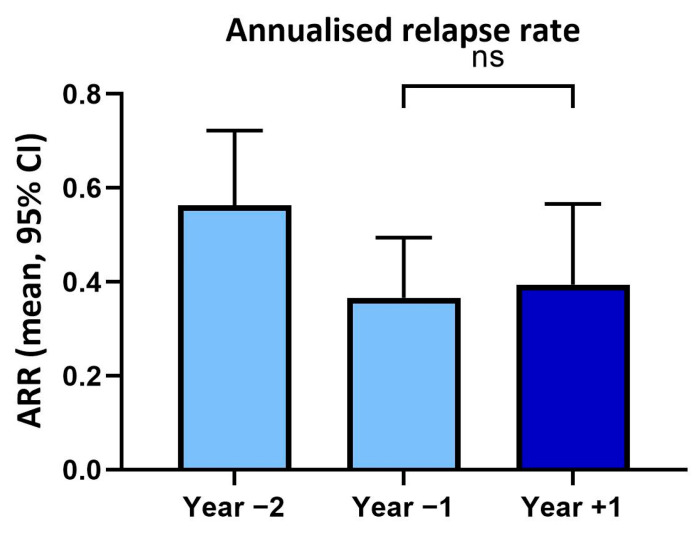
Annualised relapse rates (ARR) within two years before and one year after tetanus/diphtheria vaccination. ns: not significant.

In year two prior to vaccination, patients had an annualised relapse rate of 0.56 (SD ± 0.67) and in the year before vaccination of 0.37 (SD ± 0.54). In the year following vaccination, the annualised relapse rate (0.39, SD ± 0.77) was not significantly changed (*p* > 0.05) compared to the previous year (Figure 1).

**Figure 2:**
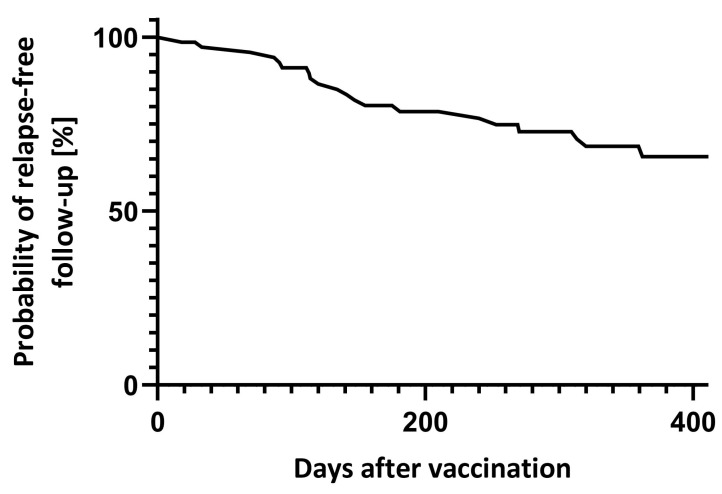
Relapse-free follow-up after vaccination.

Between day 7 and 90 after vaccination, 4 (5.6%) relapses were reported. In total, 18 patients had at least one relapse during the year after vaccination (Figure 2). Transient neurological deterioration reported on days three, four and five after vaccination in one patient each did not fulfil MS relapse criteria.

**Figure 3:**
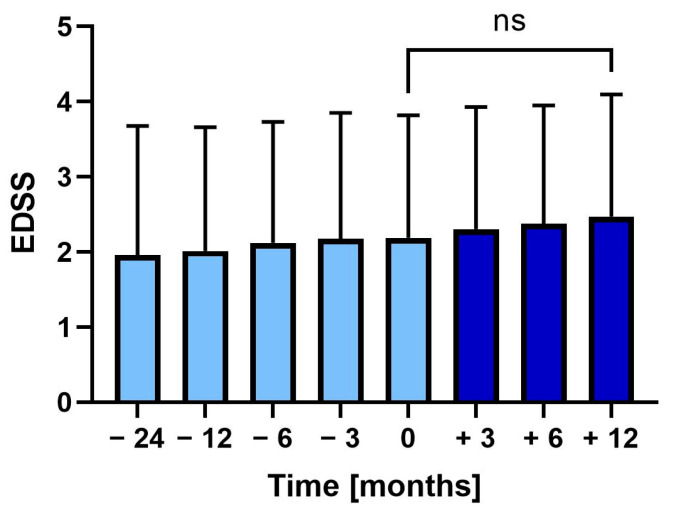
Mean Expanded Disability Status Scale (EDSS) 24 months before to 12 months after tetanus-diphtheria vaccination. Error bars: SD of mean, ns: not significant.

During the two years before vaccination, the EDSS increased from 2.0 (SD ± 1.7) to 2.2 (SD ± 1.6) at vaccination (*p* = 0.375). Following vaccination, EDSS further increased to 2.5 (SD ± 1.6), though not significantly (Figure 3).

Following vaccination, 14 (19.4%) patients had local AEFI, among them pain (*n* = 8), swelling (*n* = 4), induration (*n* = 3), erythema (*n* = 3), thermal sensation (*n* = 2). Systemic AEFI were reported by 10 (13.9%) patients, including Uhthoff’s phenomenon (*n* = 3), flu-like symptoms (*n* = 6), headache (*n* = 1) and fatigue (*n* = 1). All AEFIs were mild and resolved spontaneously within days.

**Figure 4:**
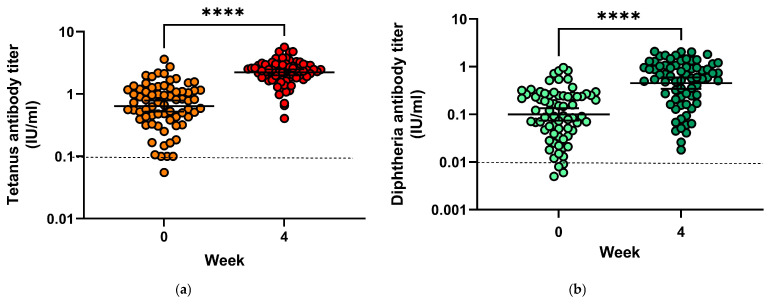
(**a**) Tetanus toxoid antibody titres before and 28 days after vaccination; (**b**) Diphtheria antibody toxoid titres before and 28 days after vaccination. **** = *p* < 0.0001.

Prior to vaccination, only four subjects (5.6%) had tetanus antibody titres below 0.1 IU/mL, indicating insufficient protection (Figure 4a), and 24 patients (33.3%) had antibody titres compatible with long-term protection (≥1 IU/mL). Geometric mean titres increased from 0.64 IU/mL (mean = 0.875 IU/mL) to 2.23 IU/mL (mean = 2.44 IU/mL) (*p* < 0.0001). Geometric mean titre ratios (GMTR) from pre-vaccination to four weeks after vaccination were 5.19. Following vaccination, all subjects had tetanus toxoid antibody titres conveying protection.

Only four subjects (5.6%) had insufficient protection against diphtheria before vaccination with antibody titres directed against diphtheria toxin below 0.01 IU/mL, and none had titres compatible with long-term protection (titres > 1.0 IU/mL) (Figure 4b). Following vaccination, all subjects had protective antibody titres, among them 19 (26.8%) developed titres above 1.0 IU/mL, 42 (59.2%) had titres between 0.1 and <1.0 IU/mL (reliable protection) and 10 (7.1%) had titres between 0.01 and <0.1 IU/mL (minimal protection). Geometric mean titres increased from 0.1 IU/mL (mean = 0.19) to 0.45 IU/mL (mean = 0.72), and GMTR from pre-vaccination to four weeks after vaccination was 8.73.

Comparing patients treated with platform therapies (beta-interferons or glatiramer acetate, *n* = 58) to patients receiving highly active treatment (natalizumab or fingolimod, *n* = 9), antibody titres against tetanus or diphtheria toxoid before vaccination did not differ between both treatment groups. Following vaccination, antibody titres against diphtheria toxoid did not differ between subjects with platform therapies and highly active treatment, whereas tetanus toxoid antibody titres were lower in subjects with highly active treatment compared to platform therapies (geometric mean: 1.68 IU/mL vs. 2.43 IU/mL, *p* < 0.01). A comparison of interferon-treated patients with glatiramer-acetate-treated patients showed significant tetanus toxoid antibody titre increase following vaccination in both groups (0.96 to 2.66 and 0.82 to 2.52, *p* < 0.0001, respectively). Mean post-vaccination titres did not differ between interferon-treated patients and glatiramer-acetate-treated patients (*p* = 0.56). In contrast, antibodies against diphtheria antigen showed a significant increase in both groups, but titres were lower in glatiramer-acetate-treated patients compared to patients treated with interferons (0.88 vs. 2.66; *p* < 0.0001).

Among patients treated with fingolimod, two of three had a significant increase [≥2-fold] in tetanus and all had a ≥2-fold increase in diphtheria toxoid antibody titres. Of the six patients treated with natalizumab, five had a significant increase [≥2-fold] in tetanus antibodies and all of these patients had a significant increase [≥2-fold] in diphtheria toxoid antibody titres. Interferon-treated patients had geometric mean titre ratios from pre-vaccination to four weeks after vaccination of 4.77 for tetanus and 8.7 for diphtheria toxoid antibodies.

## 4. Discussion

As recommended in MS guidelines, PwMS should be vaccinated against tetanus and diphtheria according to national immunisation schedules [6,10,35,36,38]. However, due to concerns about the potential for vaccinations to trigger MS-relapses, vaccine hesitancy may be present in patients and their treating physicians. This may result in reduced vaccine uptake among MS patients [44], though in some cohorts of PwMS, vaccination rates were above rates of the general population [5].

In the case of live vaccines, a small study investigating the yellow fever vaccination suggested an elevated risk of triggering relapses in MS patients [30], whereas a recent study neither showed an increase in clinical disease activity nor cerebral or spinal MRI activity [34]. Furthermore, additional studies of other live and inactivated vaccines did not show an increased risk of MS relapses [6]. With Td vaccination, two studies found no elevated risk of relapses following vaccination, though monitoring was only carried out for a short time period after vaccination [32,45].

Only patients receiving stable disease-modifying therapy, regardless of disease activity, were included to enable a reliable assessment of therapy effects on vaccine response. This approach may have resulted in a cohort with a higher prevalence of active MS disease and fewer patients on highly effective therapies. In this real-world multi-centre setting, treatment changes were not undertaken, primarily due to patient preference and, to some extent, clinical decisions by the treating neurologists. In this study, annual relapse rates did not change between the year before and the year after vaccination. Moreover, relapses occurring within 90 days following vaccination occurred in 5.6% of this cohort. In contrast, 9.3% of post-vaccination relapses were reported during the three months following SARS-CoV-2 vaccinations in a longitudinal study of MS patients [46]. Notably, relapse rates declined over the two years preceding study inclusion. EDSS did not change significantly in the year following vaccination. This was also described in MS cohorts vaccinated against seasonal influenza or tick-borne encephalitis [47,48].

Although MRI, including gadolinium-enhanced imaging, is valuable for detecting subclinical disease activity, the longstanding clinico-radiological paradox in MS [49,50,51,52] limits its standalone interpretability. Consequently, we did not assess MRI during this study prospectively; instead, we monitored safety and disease activity in a real-world setting, using standard clinical parameters like annualised relapse rate and EDSS to address the association between vaccination and disease activity in an appropriate time frame.

In this study, 19.4% of vaccinated patients reported any local and 13.9% any systemic adverse reactions following vaccination. Among the systemic reactions, three (4.2%) patients developed transient neurological deterioration with fever on days three to five, which was judged by the treating physicians to be Uhthoff’s phenomenon. In another study evaluating adverse events in younger adults, 69.9% reported local side effects and 32.5% reported systemic side effects following Td-IPV vaccination [53]. Comparable rates of injection site reactions (70.3%) were reported in another study in healthy adults vaccinated with Tdap [54]. Lower rates of AEFI in our study may be due to the older age group and possibly a longer interval to the previous tetanus-based vaccination [55,56]. Additionally, there may be a reporting bias, as adverse events were assessed retrospectively four weeks after vaccination [57,58].

In this study, all patients were eligible for vaccination with tetanus and diphtheria as they had been vaccinated 10 years or more before inclusion; previous vaccination was not documented. All had Td-based vaccination at least 10 years prior to inclusion in this study, which might contribute to the waning antibody response [59].

Individual antibody titres directed against tetanus or diphtheria only serve as surrogate markers for protection. However, antibody titres above 0.01 IU/mL are reported to be associated with protection against tetanus. Diphtheria titres above 0.01 IU/mL are considered to convey minimal protection and titres of 0.1 IU/mL and above confer full protection [42,60]. Overall, antibody titres to tetanus and diphtheria vaccination were within levels associated with protection four weeks after vaccination in this study.

Protective tetanus toxoid antibody titres were preserved five years after booster vaccination in healthy younger and older adults [61]. In our study, 94.4% of subjects had protective tetanus toxoid antibody titres before vaccination, in line with other studies evaluating booster vaccines, with 97% of participants being protected before tetanus-based booster vaccination [62,63]. Notably, we did not evaluate humoral response beyond four weeks after vaccination, although such data could have provided additional insights into the long-term effect of different DMTs on antibody responses. The focus of this study, with regard to immune response, was to compare antibody titres at an early stage following vaccination. Booster vaccination in healthy adults resulted in an increase of diphtheria toxoid antibody titres from 0.06 IU/mL to 0.45 IU/mL [64]. This was in line with our results, with mean titres rising from 0.1 IU/mL to 0.45 IU/mL in MS patients.

Td-containing vaccines mounted comparable antibody responses between DMF (dimethyl fumarate) and IFN-beta treated people with MS, and for IFN-beta treated patients, GMTR was 5.0 and 3.8 for tetanus and diphtheria, respectively [65]. GMTR in IFN-beta-treated patients was comparable to tetanus in our study (4.77), whereas for diphtheria, the GMTR was higher (8.7), though not clinically significant. A study investigating tetanus-based vaccines in MS patients randomised to treatment with ocrelizumab or interferon-beta/no therapy found sufficient antibody response in both groups, though the antibody response was lower in the ocrelizumab-treated group [14].

In PwMS treated with highly active drugs (e.g., natalizumab, fingolimod), a decreased reaction to vaccination has been described [13,66]. In this study, one of three patients treated with fingolimod and one of six patients treated with natalizumab had an antibody titre increase below two-fold after tetanus vaccination, whereas all patients had more than a two-fold increase after diphtheria vaccination.

Tetanus and diphtheria containing vaccines had a comparable antibody response in alemtuzumab-treated MS patients and matched healthy historical controls [67]. All of these MS patients had protective antibody titres prior to alemtuzumab therapy.

We acknowledge that this study primarily involves PwMS treated with platform therapies such as glatiramer acetate and beta-interferons, though highly active therapies are increasingly available. However, the platform therapies are still a relevant treatment strategy for MS therapy worldwide [68,69,70].

## 5. Conclusions

Overall, in this real-life setting, tetanus and diphtheria vaccination proved to be safe and effective in this prospective non-randomised longitudinal multicentre study in MS patients.

## Figures and Tables

**Table 1:** Characteristics of the enrolled subjects.

Group	Total
	N = 72
**Age (Years) ± SD ^1^**	39.9 ± 9.9
**Sex**	
Male	18/72 (25%)
Female	54/72 (75%)
Mean duration of disease (years) ± SD	7.7 ± 5.5 (min 0.6; max 21.5)
EDSS at vaccination ± SD	2.2 ± 1.6 (min 0; max 5.5)
**MS Disease course**	
RR-MS	68 (94%)
SP-MS	4 (6%)
**Current DMD at vaccination**	
Interferon beta	40
Interferon beta 1b	10
Interferon beta 1a s.c.	24
Interferon beta 1a i.m.	6
Glatiramer acetate	18
Fingolimod	3
Natalizumab	6
Cyclic glucocorticosteroid pulses	1
Intrathecal triamcinolone	1
Azathioprine	1
No treatment	2

^1^ SD: standard deviation.

## Data Availability

The raw data supporting the conclusions of this article will be made available by the authors on request.
